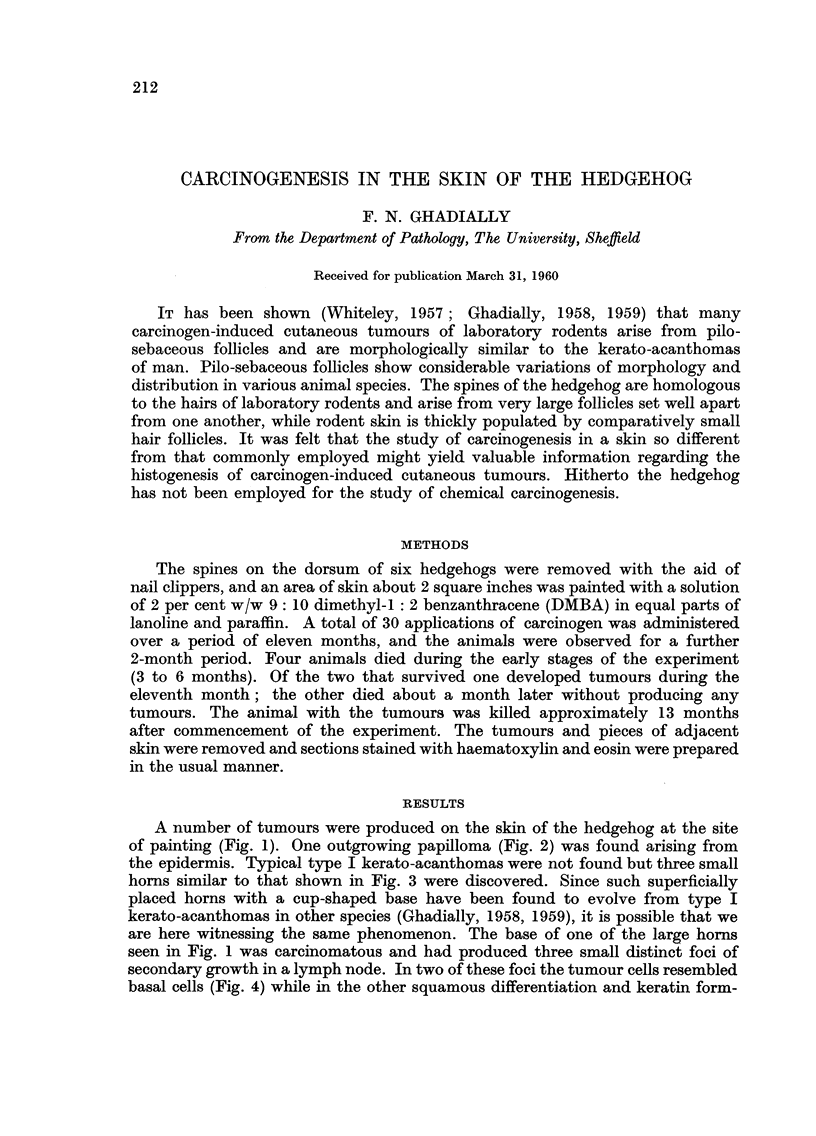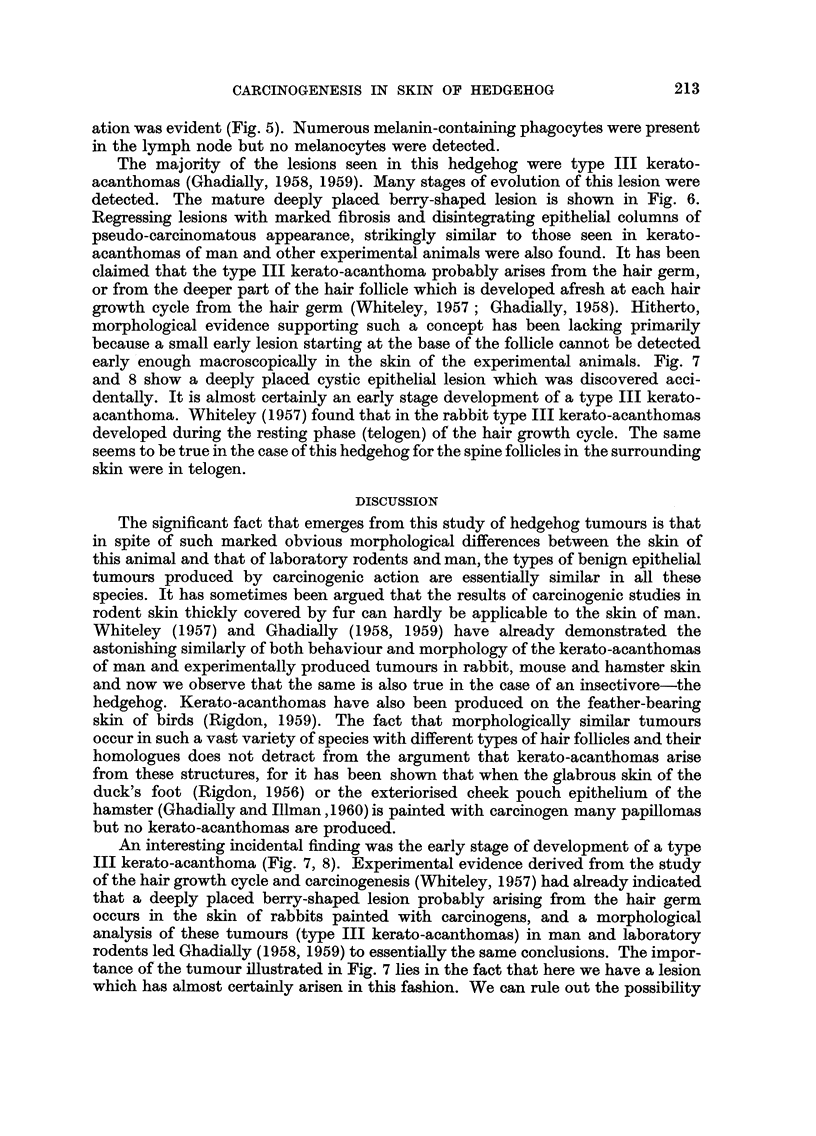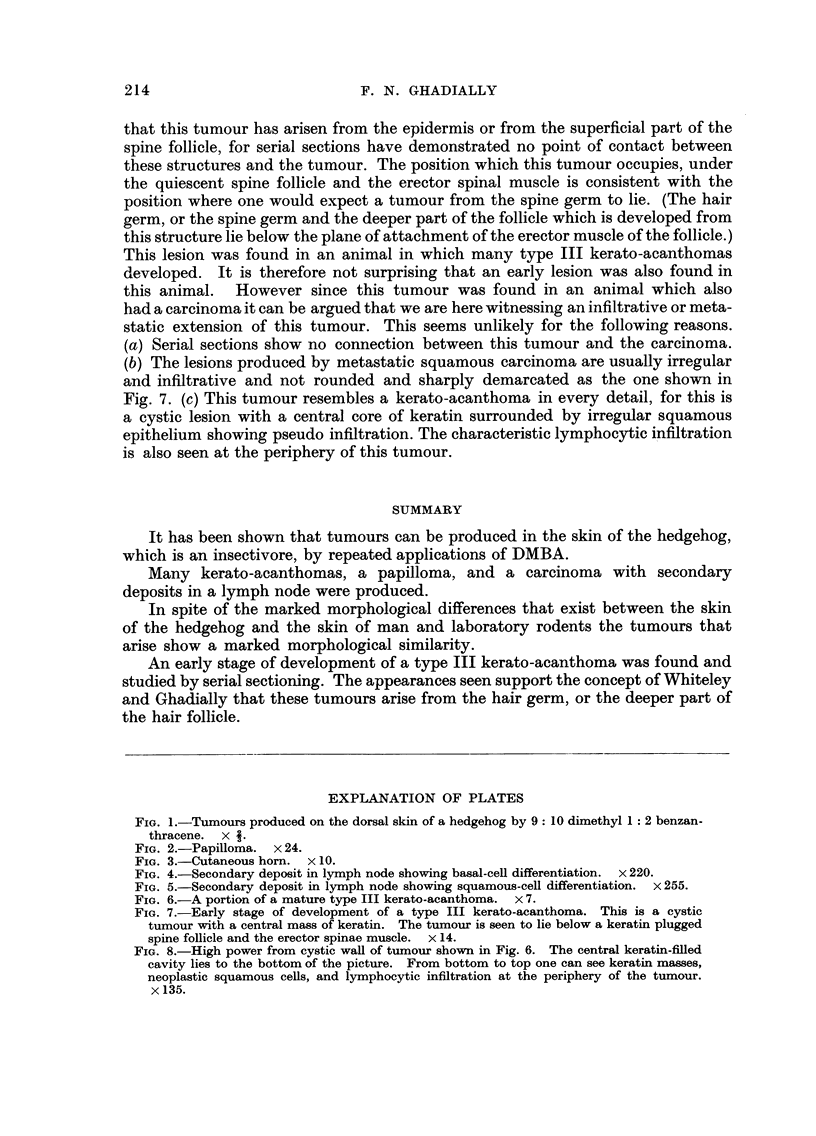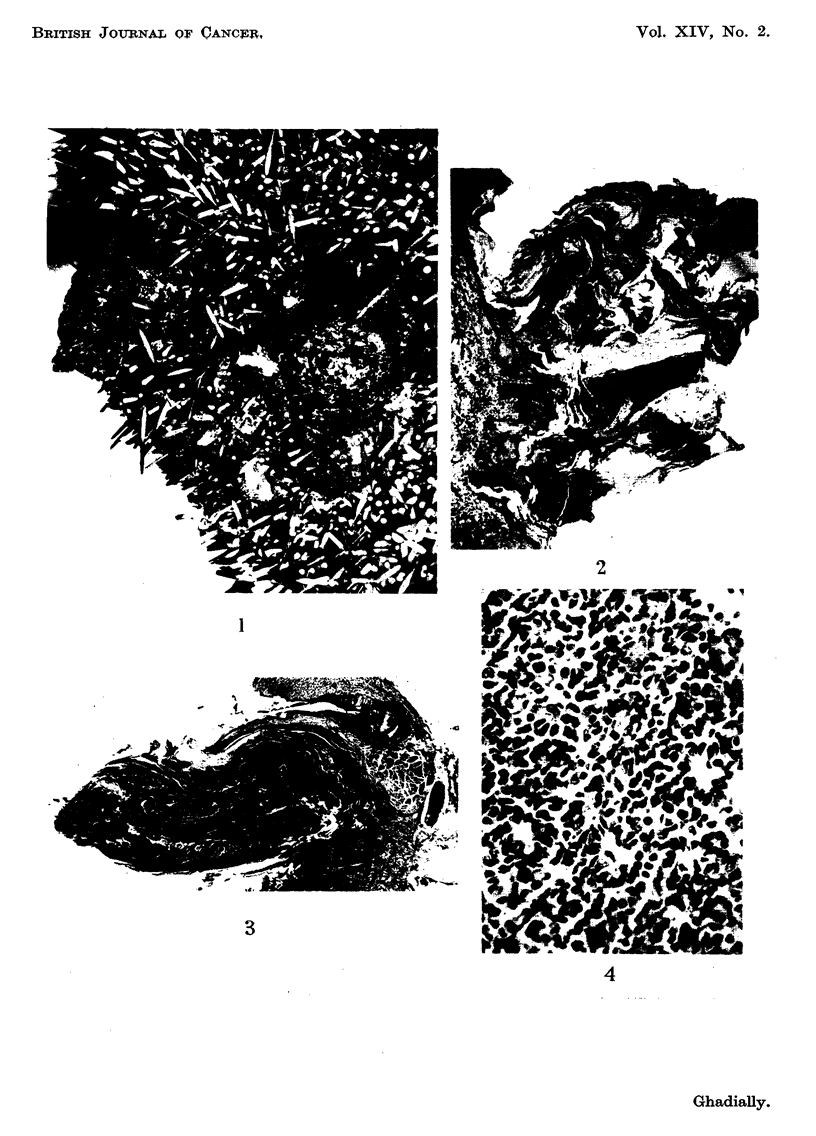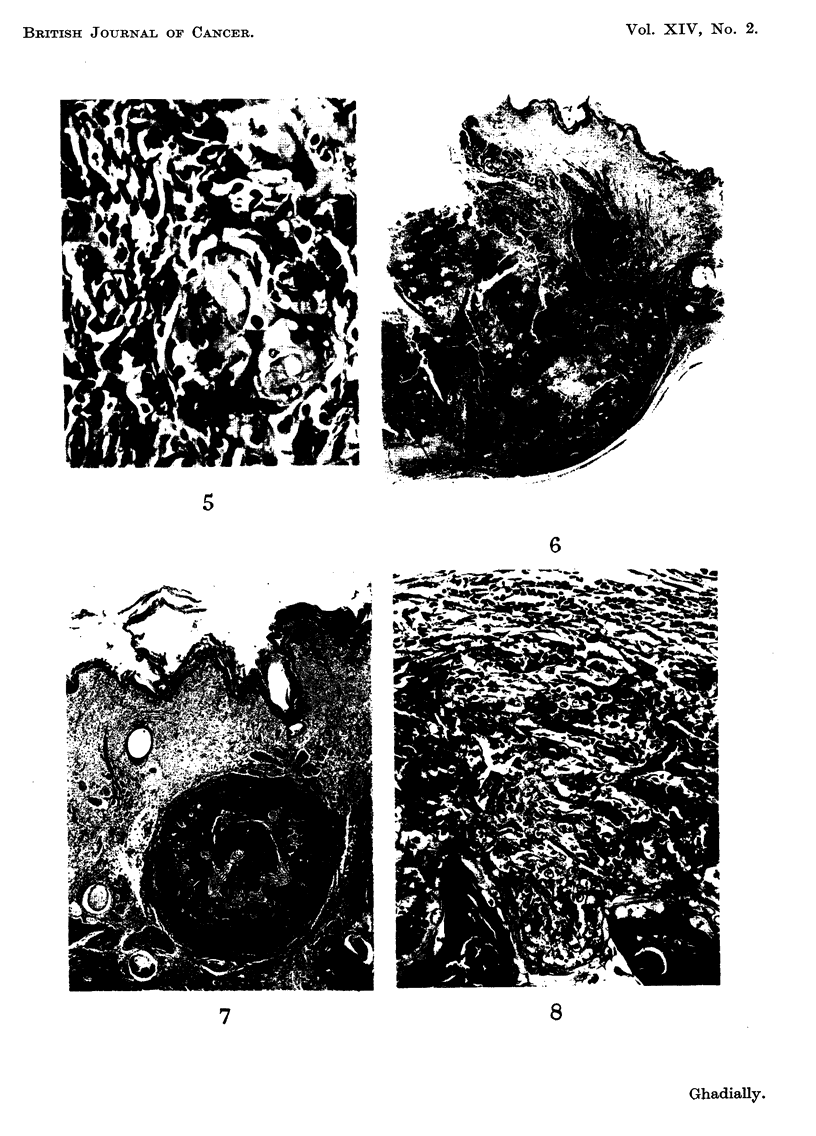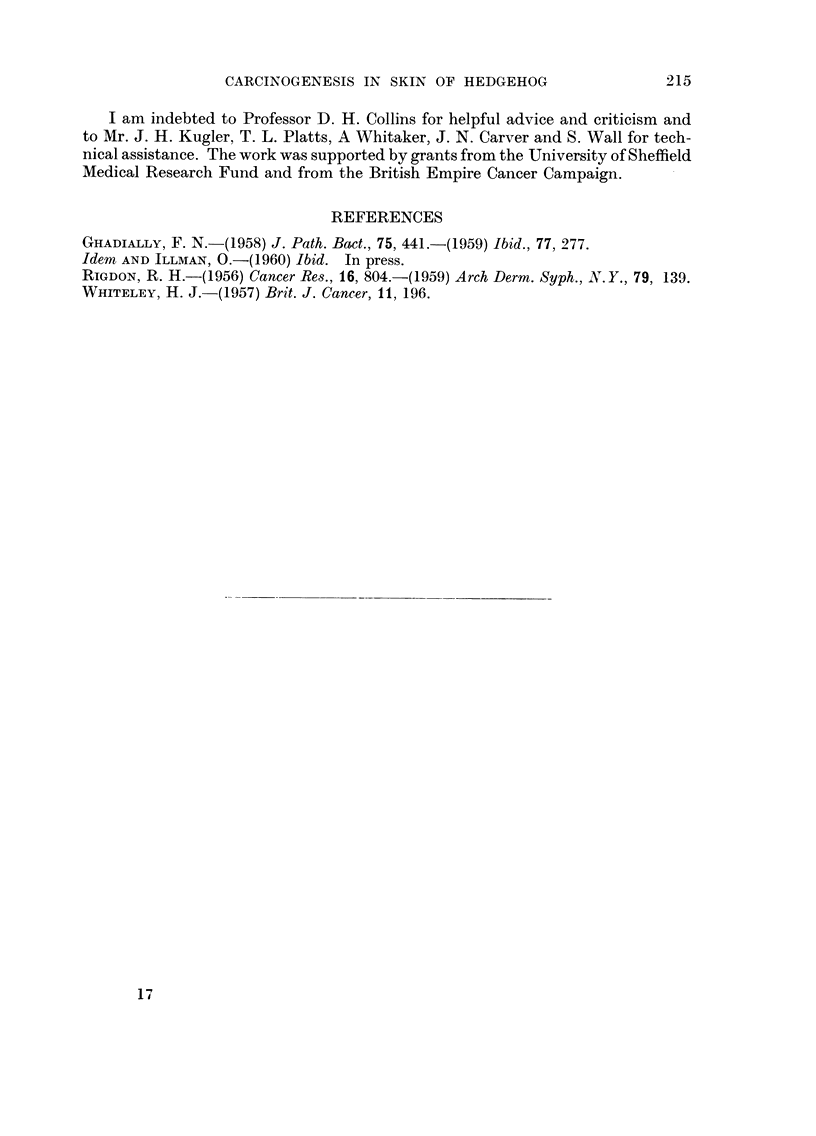# Carcinogenesis in the Skin of the Hedgehog

**DOI:** 10.1038/bjc.1960.26

**Published:** 1960-06

**Authors:** F. N. Ghadially

## Abstract

**Images:**


### CARCINOGENESIS IN THE SKIN OF THE HEDGEHOG

F. N. GHADIALLY

*From the Department of Pathology, The University, Sheffield*

Received for publication March 31, 1960

IT has been shown (Whiteley, 1957; Ghadially, 1958, 1959) that many carcinogen-induced cutaneous tumours of laboratory rodents arise from pilo-sebaceous follicles and are morphologically similar to the kerato-acanthomas of man. Pilo-sebaceous follicles show considerable variations of morphology and distribution in various animal species. The spines of the hedgehog are homologous to the hairs of laboratory rodents and arise from very large follicles set well apart from one another, while rodent skin is thickly populated by comparatively small hair follicles. It was felt that the study of carcinogenesis in a skin so different from that commonly employed might yield valuable information regarding the histogenesis of carcinogen-induced cutaneous tumours. Hitherto the hedgehog has not been employed for the study of chemical carcinogenesis.

### METHODS

The spines on the dorsum of six hedgehogs were removed with the aid of nail clippers, and an area of skin about 2 square inches was painted with a solution of 2 per cent w/w 9 : 10 dimethyl-1 : 2 benzanthracene (DMBA) in equal parts of lanoline and paraffin. A total of 30 applications of carcinogen was administered over a period of eleven months, and the animals were observed for a further 2-month period. Four animals died during the early stages of the experiment (3 to 6 months). Of the two that survived one developed tumours during the eleventh month; the other died about a month later without producing any tumours. The animal with the tumours was killed approximately 13 months after commencement of the experiment. The tumours and pieces of adjacent skin were removed and sections stained with haematoxylin and eosin were prepared in the usual manner.

### RESULTS

A number of tumours were produced on the skin of the hedgehog at the site of painting (Fig. 1). One outgrowing papilloma (Fig. 2) was found arising from the epidermis. Typical type I kerato-acanthomas were not found but three small horns similar to that shown in Fig. 3 were discovered. Since such superficially placed horns with a cup-shaped base have been found to evolve from type I kerato-acanthomas in other species (Ghadially, 1958, 1959), it is possible that we are here witnessing the same phenomenon. The base of one of the large horns seen in Fig. 1 was carcinomatous and had produced three small distinct foci of secondary growth in a lymph node. In two of these foci the tumour cells resembled basal cells (Fig. 4) while in the other squamous differentiation and keratin formation was evident (Fig. 5). Numerous melanin-containing phagocytes were present in the lymph node but no melanocytes were detected.

The majority of the lesions seen in this hedgehog were type III kerato-acanthomas (Ghadially, 1958, 1959). Many stages of evolution of this lesion were detected. The mature deeply placed berry-shaped lesion is shown in Fig. 6. Regressing lesions with marked fibrosis and disintegrating epithelial columns of pseudo-carcinomatous appearance, strikingly similar to those seen in kerato-acanthomas of man and other experimental animals were also found. It has been claimed that the type III kerato-acanthoma probably arises from the hair germ, or from the deeper part of the hair follicle which is developed afresh at each hair growth cycle from the hair germ (Whiteley, 1957 ; Ghadially, 1958). Hitherto, morphological evidence supporting such a concept has been lacking primarily because a small early lesion starting at the base of the follicle cannot be detected early enough macroscopically in the skin of the experimental animals. Fig. 7 and 8 show a deeply placed cystic epithelial lesion which was discovered accidentally. It is almost certainly an early stage development of a type III kerato-acanthoma. Whiteley (1957) found that in the rabbit type III kerato-acanthomas developed during the resting phase (telogen) of the hair growth cycle. The same seems to be true in the case of this hedgehog for the spine follicles in the surrounding skin were in telogen.

### DISCUSSION

The significant fact that emerges from this study of hedgehog tumours is that in spite of such marked obvious morphological differences between the skin of this animal and that of laboratory rodents and man, the types of benign epithelial tumours produced by carcinogenic action are essentially similar in all these species. It has sometimes been argued that the results of carcinogenic studies in rodent skin thickly covered by fur can hardly be applicable to the skin of man. Whiteley (1957) and Ghadially (1958, 1959) have already demonstrated the astonishing similarly of both behaviour and morphology of the kerato-acanthomas of man and experimentally produced tumours in rabbit, mouse and hamster skin and now we observe that the same is also true in the case of an insectivore—the hedgehog. Kerato-acanthomas have also been produced on the feather-bearing skin of birds (Rigdon, 1959). The fact that morphologically similar tumours occur in such a vast variety of species with different types of hair follicles and their homologues does not detract from the argument that kerato-acanthomas arise from these structures, for it has been shown that when the glabrous skin of the duck's foot (Rigdon, 1956) or the exteriorised cheek pouch epithelium of the hamster (Ghadially and Illman ,1960) is painted with carcinogen many papillomas but no kerato-acanthomas are produced.

An interesting incidental finding was the early stage of development of a type III kerato-acanthoma (Fig. 7, 8). Experimental evidence derived from the study of the hair growth cycle and carcinogenesis (Whiteley, 1957) had already indicated that a deeply placed berry-shaped lesion probably arising from the hair germ occurs in the skin of rabbits painted with carcinogens, and a morphological analysis of these tumours (type III kerato-acanthomas) in man and laboratory rodents led Ghadially (1958, 1959) to essentially the same conclusions. The importance of the tumour illustrated in Fig. 7 lies in the fact that here we have a lesion which has almost certainly arisen in this fashion. We can rule out the possibility that this tumour has arisen from the epidermis or from the superficial part of the spine follicle, for serial sections have demonstrated no point of contact between these structures and the tumour. The position which this tumour occupies, under the quiescent spine follicle and the erector spinal muscle is consistent with the position where one would expect a tumour from the spine germ to lie. (The hair germ, or the spine germ and the deeper part of the follicle which is developed from this structure lie below the plane of attachment of the erector muscle of the follicle.) This lesion was found in an animal in which many type III kerato-acanthomas developed. It is therefore not surprising that an early lesion was also found in this animal. However since this tumour was found in an animal which also had a carcinoma it can be argued that we are here witnessing an infiltrative or metastatic extension of this tumour. This seems unlikely for the following reasons. (*a*) Serial sections show no connection between this tumour and the carcinoma. (*b*) The lesions produced by metastatic squamous carcinoma are usually irregular and infiltrative and not rounded and sharply demarcated as the one shown in Fig. 7. (*c*) This tumour resembles a kerato-acanthoma in every detail, for this is a cystic lesion with a central core of keratin surrounded by irregular squamous epithelium showing pseudo infiltration. The characteristic lymphocytic infiltration is also seen at the periphery of this tumour.

### SUMMARY

It has been shown that tumours can be produced in the skin of the hedgehog, which is an insectivore, by repeated applications of DMBA.

Many kerato-acanthomas, a papilloma, and a carcinoma with secondary deposits in a lymph node were produced.

In spite of the marked morphological differences that exist between the skin of the hedgehog and the skin of man and laboratory rodents the tumours that arise show a marked morphological similarity.

An early stage of development of a type III kerato-acanthoma was found and studied by serial sectioning. The appearances seen support the concept of Whiteley and Ghadially that these tumours arise from the hair germ, or the deeper part of the hair follicle.

---

### EXPLANATION OF PLATES

FIG. 1.—Tumours produced on the dorsal skin of a hedgehog by 9 : 10 dimethyl 1 : 2 benzanthracene. $\times \frac{2}{3}$.
FIG. 2.—Papilloma. $\times 24$.
FIG. 3.—Cutaneous horn. $\times 10$.
FIG. 4.—Secondary deposit in lymph node showing basal-cell differentiation. $\times 220$.
FIG. 5.—Secondary deposit in lymph node showing squamous-cell differentiation. $\times 255$.
FIG. 6.—A portion of a mature type III kerato-acanthoma. $\times 7$.
FIG. 7.—Early stage of development of a type III kerato-acanthoma. This is a cystic tumour with a central mass of keratin. The tumour is seen to lie below a keratin plugged spine follicle and the erector spinae muscle. $\times 14$.
FIG. 8.—High power from cystic wall of tumour shown in Fig. 6. The central keratin-filled cavity lies to the bottom of the picture. From bottom to top one can see keratin masses, neoplastic squamous cells, and lymphocytic infiltration at the periphery of the tumour. $\times 135$.

1

2

3

4

5

6

7

8

Ghadially.

I am indebted to Professor D. H. Collins for helpful advice and criticism and to Mr. J. H. Kugler, T. L. Platts, A Whitaker, J. N. Carver and S. Wall for technical assistance. The work was supported by grants from the University of Sheffield Medical Research Fund and from the British Empire Cancer Campaign.

### REFERENCES

GHADIALLY, F. N.—(1958) *J. Path. Bact.*, **75**, 441.—(1959) *Ibid.*, **77**, 277.
*Idem* AND ILLMAN, O.—(1960) *Ibid.* In press.
RIGDON, R. H.—(1956) *Cancer Res.*, **16**, 804.—(1959) *Arch Derm. Syph., N.Y.*, **79**, 139.
WHITELEY, H. J.—(1957) *Brit. J. Cancer*, **11**, 196.